# Predicting discharge to institutional long-term care following acute hospitalisation: a systematic review and meta-analysis

**DOI:** 10.1093/ageing/afx047

**Published:** 2017-06-20

**Authors:** Jennifer Kirsty Harrison, Katherine E Walesby, Lorna Hamilton, Carolyn Armstrong, John M Starr, Emma L Reynish, Alasdair M J MacLullich, Terry J Quinn, Susan D Shenkin

**Affiliations:** 1 Alzheimer Scotland Dementia Research Centre, University of Edinburgh, Edinburgh, UK; 2 Centre for Cognitive Ageing and Cognitive Epidemiology, University of Edinburgh, Edinburgh, UK; 3 Department of Medicine for the Elderly, NHS Lothian, Edinburgh, UK; 4 School of Applied Social Science, University of Stirling, Stirling, UK; 5 Geriatric Medicine, University of Edinburgh, Edinburgh, UK; 6 Institute of Cardiovascular and Medical Sciences, University of Glasgow, Glasgow, UK

**Keywords:** long-term care, hospitalisation, care home, outcome, predictor, older people

## Abstract

**Background:**

moving into long-term institutional care is a significant life event for any individual. Predictors of institutional care admission from community-dwellers and people with dementia have been described, but those from the acute hospital setting have not been systematically reviewed. Our aim was to establish predictive factors for discharge to institutional care following acute hospitalisation.

**Methods:**

we registered and conducted a systematic review (PROSPERO: CRD42015023497). We searched MEDLINE; EMBASE and CINAHL Plus in September 2015. We included observational studies of patients admitted directly to long-term institutional care following acute hospitalisation where factors associated with institutionalisation were reported.

**Results:**

from 9,176 records, we included 23 studies (*n* = 354,985 participants). Studies were heterogeneous, with the proportions discharged to a care home 3–77% (median 15%). Eleven studies (*n* = 12,642), of moderate to low quality, were included in the quantitative synthesis. The need for institutional long-term care was associated with age (pooled odds ratio (OR) 1.02, 95% confidence intervals (CI): 1.00–1.04), female sex (pooled OR 1.41, 95% CI: 1.03–1.92), dementia (pooled OR 2.14, 95% CI: 1.24–3.70) and functional dependency (pooled OR 2.06, 95% CI: 1.58–2.69).

**Conclusions:**

discharge to long-term institutional care following acute hospitalisation is common, but current data do not allow prediction of who will make this transition. Potentially important predictors evaluated in community cohorts have not been examined in hospitalised cohorts. Understanding these predictors could help identify individuals at risk early in their admission, and support them in this transition or potentially intervene to reduce their risk.

## Introduction

### Rationale

A significant proportion (2–5%) of the adult population worldwide receive 24-hour care in an institutional setting such as a nursing home [1]. Moving into institutional care is a major decision, with significant personal and economic implications [2]. Admission can occur from the community, rehabilitation or intermediate care setting, or from the acute hospital. Pathways into 24-hour care—and the care provided in these settings—differ worldwide; in the UK, for example, health and social care policy and guidelines discourage direct discharge from the acute hospital [3, 4]. Hospital admission has been associated as contributing to premature admission into long-term care [5, 6], although the reasons for this have not been explored.

In population studies of older adults, predictors of institutional care admission include age, low self-rated health, functional impairment, dementia, prior nursing home placement and polypharmacy [7]. In community-dwellers with dementia, caregiver burden and dependence in activities of daily living (ADL) are established predictors [8], with the most recent data identifying associations between poorer cognitive function and behavioural and psychological symptoms of dementia (BPSD) [9]. It is not known whether these factors are the same or different in people admitted directly following an acute hospitalisation.

An acute hospital admission may occur in response to an acute illness, as a complication or progression of a chronic health condition or a deterioration in an individual's social circumstances requiring urgent help. There is nearly 4-fold national variation in rates of emergency admissions among older adults [10] and 6-fold variation in the likelihood of being admitted to a care home at hospital discharge [11]. Acute hospitals are under pressure to shorten length of stays and avoid delays associated with complex discharges [12]. However, specialist models of in-hospital care, such as receiving comprehensive geriatric assessment, have been shown to reduce the need for institutional care at discharge [13]. Identifying the predictors of long-term care admission directly from the acute hospital setting has the potential to help in service planning; to identify targets for an intervention to prevent admission; to allow benchmarking of services between regions; and to support those experiencing this transition.

### Objectives

Our aim was to perform a systematic review of predictive factors for a new admission to long-term institutional care (‘care home’) following unscheduled admission to acute medical, surgical or older adult hospital care.

## Methods

This review was reported in accordance with the Preferred Reporting of Items in Systematic Reviews and Meta-Analyses (PRISMA) guidance [14].

### Protocol and registration

The protocol was prospectively registered on 20/8/15: (CRD42015023497; http://www.crd.york.ac.uk/PROSPERO/display_record.asp?ID=CRD42015023497).

### Eligibility criteria

Studies were eligible for inclusion if they were observational, included participants who had an acute hospitalisation, to medical, surgical or older adult care wards and included any quantitative description of factors associated with care home admission. Studies of specialised hospital populations defined by a single condition or diagnosis (including stroke, trauma, haemodialysis and heart failure) were excluded. The exposure of interest was any predictive factor for long-term care admission. We were interested in the natural distribution of the predictive characteristics in the population and, as such, intervention studies seeking to alter rates of admission were excluded.

The outcome of interest was admission directly to a long-term institutional care setting (henceforth described as a ‘care home’) as new place of residence at discharge. We recognise the international heterogeneity in terminology, so defined this in an inclusive way [15]. We excluded those discharged from rehabilitation settings or those who were admitted to a care home after an interval following hospitalisation, or where care home admission was evaluated at a fixed follow-up point after discharge.

No restrictions were made on date or language of publication. If abstracts were identified, we searched for subsequent full-text publications, and contacted the authors.

### Information sources

On 28/9/15 we searched: Ovid MEDLINE (R) In Process and Other Non-Indexed Citations and Ovid MEDLINE (R) 1946 to present; Ovid EMBASE 1980 to 2015 Week 39 and EBSCOhost CINAHL Plus.

### Search

We developed the search with an Information Specialist. Results from a scoping review in 2014 were used to identify relevant Medical Subject Headings (MeSH) terms and keywords [16]. The full search strategy is included in Supplementary data, Appendix 1, are available at *Age and Ageing* online. This was supplemented by review of reference lists from identified systematic reviews.

### Study selection and data collection

Two authors (J.K.H. and K.E.W.) independently screened all titles and abstracts, then reviewed full texts for eligibility, using Covidence software [17]. Conflicts were resolved by discussion with a third author (S.D.S.). A data extraction proforma was developed and piloted to improve usability. Data extraction was performed by a single author (J.K.H.) with a two co-authors (K.E.W. and S.D.S.) performing double-extraction on a random sample of 25%. A full list of data items extracted is included in Supplementary data, text, are available in *Age and Ageing* online.

### Risk of bias assessment in individual studies

Risk of bias assessment was performed based on the Risk of Bias Assessment Tool for Non-Randomized Studies (RoBANS) [18]. Guidance was provided to co-authors to facilitate a consistent approach, provided in Supplementary data, Appendix 2, are available in *Age and Ageing* online.

### Summary measures

Studies were included if they reported quantitative data with associated statistical tests of association. These included reporting of risk ratios (RRs), odds ratios (ORs), correlations and differences in proportion between two groups with comparative significance testing.

### Synthesis of results

Quantitative analysis was performed using Comprehensive Meta-Analysis software [19]. We calculated summary estimates where data were reported on the same predictor variable from three or more studies. We used Random Effects models to calculate pooled ORs and 95% confidence intervals (CI). These data were evaluated using the Grading of Recommendations Assessment, Development and Evaluation (GRADE) approach to describe the quality of the evidence [20]. As our question of interest was to determine factors associated with care home admission, a question which can be explored using observational data, we did not downgrade the quality of the evidence due to observational study design, but for the other recognised parameters which reduce the quality of the body of evidence on a topic, including risk of bias, heterogeneity and inconsistency [20]. Statistical heterogeneity was quantified using *I*^2^ and supplemented by evaluation of the clinical heterogeneity and inspection of forest plots.

### Additional analyses

Planned sub-group analyses included residential versus nursing care; country of origin; age <65 versus ≥65; timing of assessment of predictor; dementia and delirium.

## Results

### Study selection

**Figure 1.**
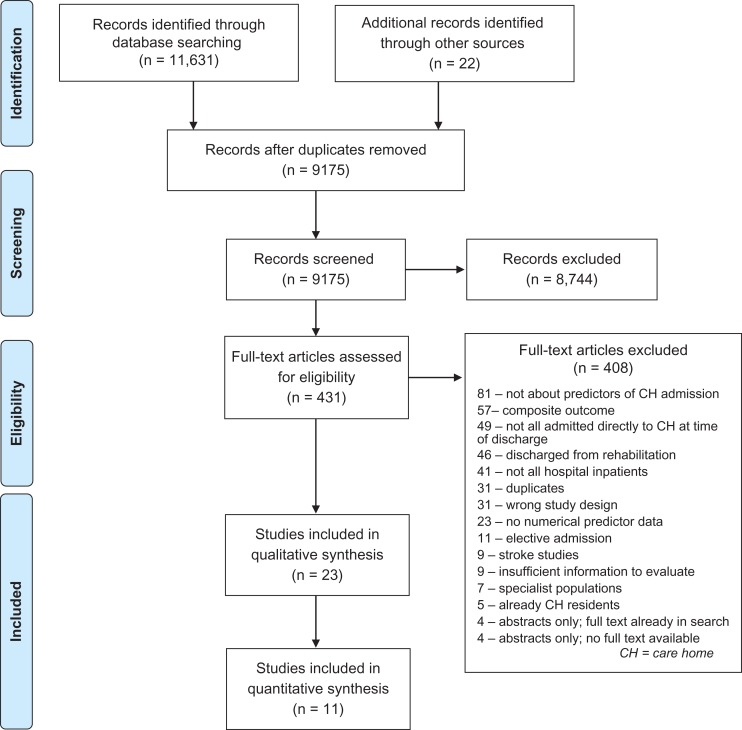
PRISMA flow diagram. [57]

The initial search identified 9,176 records after initial deduplication. Following title and abstract screening, 431 records remained for full-text review and 23 studies were included in the review [21–43]. (Figure 1).

### Included study characteristics

The total review population included 354,985 participants, from studies in Europe, North America and Hong Kong. The sample size varied significantly from 94 to 262,345 participants (median sample size 727; inter-quartile range [IQR] 1,708). A detailed summary of the characteristics of the included studies is provided in Table 1 and Supplementary data, Table 1, available in *Age and Ageing* online. Study duration was highly varied ranging from 3 months to 10 years (median duration 16 months; IQR 19 months). Two studies were translated from the Spanish text [26, 30].

**Table 1.** Summary characteristics of included studies

Study ID/year	*N*	Country	Study design	Design	Duration	Setting
Ackroyd-Stolarz 2009 [21]	982	Canada	Retrospective	Cohort study using administrative data	9 months	Acute inpatients >65 years who had no acute admissions or ED attendances in prior 6 months
Adamis 2006 [22]	94	UK	Prospective	Cohort study	3 months	Acute admissions to Elderly Care Unit (≥70 years)
Alarcon 1999 [23]	353	Spain	Prospective	Cohort study	10 months	Acute geriatric ward admissions
Astell 2008 [24]	234	UK	Prospective	Cohort study	4 years	Joint geriatric medicine/old age psychiatry unit
Basic 2015 [25]	2,125	Australia	Prospective	Cohort study	3.5 years	Tertiary referral hospital; admitted under the care of a geriatrician
Baztan 2004 [26]	459	Spain	Prospective	Cohort study	19 months	Consecutive admissions with functional disability
Bonneyfoy 1998 [27]	1,066	France	Prospective	Cohort study	26 months	Admissions to acute geriatrics unit
Bourdel-Marchasson 2004 [28]	427	France	Prospective	Cohort study	1 year	Admissions to acute care geriatric unit
Brown 2012 [29]	392	UK	Prospective	Cohort study	6 months	Admission to acute care geriatric ward
Cabre 2004 [30]	585	Spain	Prospective	Cohort study	28 months	Admissions to an acute geriatric unit
Corsinovi 2009 [31]	620	Italy	Prospective	Cohort study	16 months	Admission to geriatric acute care ward
Gordon 1995 [32]	40,820	USA	Retrospective	Cohort study	40 months	Consecutive discharges from medicine and surgery
Inouye 1998 [33]	727	USA	Prospective	Cohort study	5–8 months	Three university affiliated teaching hospitals; admissions of older adults
Isaia 2010 [62]	123	Italy	Prospective	Cohort study	8 months	Admission to the department of geriatric medicine in university hospital
Jonsson 2008 [35]	749	Nordic countries	Prospective	Cohort study	15 months	Adults ≥75 years admitted to acute medical care
Kozyrskyj 2005 [36]	17,984	Canada	Retrospective	Cohort study	7 years	Older adults in medicine and surgery with long-stay (>30 days) admission
Luk 2009 [37]	535	Hong Kong	Retrospective	Cohort study	27 months	Admissions to geriatric units
Marengoni 2008 [38]	830	Italy	Prospective	Cohort study	22 months	Consecutive admissions to acute care geriatrics
Romero-Ortuno 2014 [39]	15,873	Ireland	Retrospective	Hospital-based registry	10 years	Medical admissions aged ≥65 years
Smith 2009 [40]	6,006	USA	Retrospective	Cohort study	1 year	Discharges from those aged ≥60 years admitted to S&W Healthcare in Temple
Van Nes 2001 [41]	1,145	Switzerland	Prospective	Cohort study	2 years	Convenience sample of patients admitted to Geriatric Medicine hospital
Wong 2010 [42]	262,439	The Netherlands	Retrospective	Cohort study	1 year	Individuals aged ≥65 years, admitted to hospital not utilising any kind of formal care
Zureik 1995 [43]	417	France	Prospective	Cohort study	4 months	Admissions from home to acute medical care units of individuals aged ≥75

The proportion of included participants who were discharged to a care home varied from 3 to 77% (median 15%, IQR 17). In three studies it was not possible to calculate the proportion discharged to a care home from the data reported [31, 34, 39]. Only two of the studies were not aimed at determining predictors of care home admission [34, 39].

There was significant heterogeneity in terminology used to describe the setting and none of the included studies operationalised a definition of ‘care home’ setting.

### Risk of bias within studies

The risk of bias assessment is summarised in Supplementary data, Figure 1, are available in *Age and Ageing* online. No study was considered at low risk of bias in all domains. Issues of concern were around the selection of participants, often with recruitment of non-consecutive samples and restrictive exclusion criteria. Only one study had a protocol allowing for evaluation of selective outcome reporting.

### Quantitative results

The 23 studies adopted a range of approaches to determine predictors. Determining the association of care home admission with cognition, functional performance and mobility was complicated by the inconsistency in how these factors were assessed and described, even predictors such as age or length of stay were often categorised differently.

#### Studies using multivariate analyses

The majority of included papers (13/23) presented multivariate models with predictors, summarised in Table 2. Seven papers presented strength of association for all variables of interest including those where no statistically significant association was identified [25, 26, 28, 35, 38, 42, 43].

**Table 2.** Multivariate predictors of care home admission from general and geriatric medicine studies

Study ID	Potential predictors evaluated	Statistically significant predictors		
		Predictor		OR or RR
Ackroyd [21]	Age; sex; co-morbidities; length of stay Other: use of ventilator; occurrence of an adverse event	Length of stay (days)		OR 1.04 (1.02–1.06)
Alarcon[23]	Living alone; cognitive impairment; malnutrition; functional ability; polypharmacy Other: pressure sores; pesence of a pension; family carer	Functional disability (measured using the Red Cross Functional Disability Scale >3)		OR 3.41 (1.46–5.00)
Basic [25]	Age; dementia; delirium; co-morbidities Other: frailty; urinary retention; deconditioning	Dementia		D: OR 1.83 (1.00–3.37); V: 2.06 (1.19–3.55)
		Frailty		D: OR 2.08 (1.40–3.10) V: OR 1.60 (1.14–2.24)
		Urinary retention		D: 2.60 (1.23–5.47); V: 3.30 (1.77–6.13)
		Deconditioning		D: 2.93 (1.64–5.23); V: 2.57 (1.53–4.32)
Baztan [26]	Age; sex; cognitive impairment; admission diagnosis; functional status; co-morbidities Other: albumin; sociofamiliar scale	Age (years)		OR 1.06 (1.01–1.12)
		Admission diagnosis (orthopaedic versus not)		OR 1.06 (1.01–1.12)
		Other (sociofamiliar scale ≥9)		OR 6.83 (1.91–24.47)
Bourdel-Marchasson [28]	Age; sex; delirium; cognitive impairment; malnutrition; polypharmacy; comorbidity; function; admission diagnosis Other: weight	Sex (female)		OR 2.15 (1.22–3.78)
		Prevalent delirium (CAM)		OR 3.19 (1.33–7.64)
		Malnutrition (low intake)		OR 2.5 (1.35–4.63)
		Admission diagnosis		
		Falls Stroke		OR 2.16 (1.22–3.84) OR 2.03 (1.04–3.94)
Cabre [30]	Age; dementia; delirium; malnutrition; mobility; functional ability; co-morbidities; incontinence Other: sleep disorder; pressure ulcers; falls	Mobility (reduced)		OR 3.1 (1.69–5.67)
		Function Barthel Index (0–20 versus >60)		OR 3.19 (1.34–7.58)
		Barthel Index (21–40 versus >60)		OR 3.6 (1.51–8.59)
		Co-morbidities Cancer		OR 0.28 (0.08–0.97)
		Chronic lung disease		OR 0.50 (0.29–0.89)
		Other: falls (in last year)		OR 2.99 (1.78 –5.00)
Jonsson [35]	Age; sex; cognitive impairment; functional ability; prior care; admission diagnoses Other: country	Cognitive impairment (moderate/severe on cognitive performance scale)		OR 8.63 (3.91–19.01)
		Functional ability (Problems with IADLs)		OR 6.04 (1.35–27.12)
Kozyrskyj [36]	Living alone; cognitive impairment; admission diagnosis; co-morbidities; length of admission; prior care; Other: income; in-hospital fall; winnipeg resident; surgical versus medical; geriatric unit; dialysis; rehabilitation; discharge hospital; year; other diagnoses *Results presented stratified by age group: 65*–*74; 75*–*84; ≥85*	Living alone (65–74 only)		OR 1.27 (1.08–1.48)
		Cognitive impairment 65–74		OR 2.42 (1.65–3.56)
		75–84		OR 2.75 (2.16–3.50)
		≥85		OR 1.51 (1.20–1.90)
		Admission diagnosis: stroke 65–74		OR 1.83 (1.33–2.53)
		75–84		OR 1.93 (1.59–2.33)
		≥85		OR 1.54 (1.29–1.86)
		Admission diagnosis: nervous system disorder 65–74		OR 2.08 (1.21–3.57)
		75–84		OR 3.05 (2.08–4.46)
		≥85		OR 1.72 (1.08–2.75)
		Co-morbidities (using Charlson Index) 65–74 Some		OR 1.33 (1.04–1.69)
		75–84 Multiple		OR 0.73 (0.56–0.95)
		≥85 Multiple		OR 0.68 (0.49–0.94)
		Length of admission (>120 days) 65–74		OR 6.65 (5.10–8.67)
		75–84		OR 7.16 (6.05–8.46)
		≥85		OR 2.05 (1.70–2.47)
		Prior home care 65–74		OR 1.55 (1.31–1.83)
		75–84		OR 1.48 (1.34–1.62)
		≥85		OR 1.40 (1.27–1.54)
		Lowest income 75–84		OR 1.18 (1.01–1.37)
		≥85		OR 1.23 (1.07–1.42)
		In-hospital		
		Fall 75–84 No fall ≥85		OR 1.25 (1.01–1.55) OR 7.00 (5.78–8.48)
Luk [37]	Age; cognition; mobility; admission diagnosis; functional status; length of stay Other: pressure sores; marital status; albumin level	Cognitive performance (Higher C-MMSE)		OR 0.93 (0.87–0.98)
		Urinary incontinence		OR 5.13 (2.66–10.6)
		Mobility (Higher Elderly Mobility Scale)		OR 0.91 (0.84–0.97)
		Admission diagnosis: Falls		OR 2.4 (1.03–5.57)
		Marital status		OR 2.74 (1.36–5.53)
		Albumin level (Higher; level not stated)		OR 0.93 (0.88–0.99)
Marengoni [38]	Age; sex; cognitive impairment; functional ability; co-morbidities; length of admission; living alone other: education; presence of a caregiver	Functional ability (BADLs) (continuous variable)		OR 1.4 (1.1–1.9)
		Length of admission (Days)		OR 1.1 (1.0–1.1)
Smith [40]	Sex; living alone; functional ability; mobility; prior care other: behaviour; sleep; weight change; readiness to learn; pain; readmission; risk of mortality; race; falls risk; understanding of illness; abnormal affect; impaired level of consciousness; presence of caregiver	Sex (male versus female)		OR 1.5 (1.26–1.77)
		Living alone		OR 1.75 (1.43–2.14)
		Functional ability (help with dressing)		OR 1.63 (1.34–1.98)
		Falls risk		OR 2.25 (1.78–2.84)
		Understanding of illness		OR 2.07 (1.58–2.71)
		Abnormal affect		OR 1.80 (1.36–2.38)
		Impaired level of consciousness		OR 1.76 (1.31–2.32)
		Presence of caregiver		OR 0.76 (0.65–0.97)
		Other: education		OR 0.74 (0.58–0.94)
Wong [42]*	Age; sex; dementia; admission diagnosis; length of admission Other: presence of spouse; presence of child	Age (years)		RRR 1.34
		Dementia		RRR 7.50
		Admission diagnosis	Gastrointestinal cancer	RRR 1.25
			Lung cancer	RRR 2.22
			Bladder cancer	RRR 0.51
			Schizophrenia	RRR 3.89
			Epilepsy	RRR 1.33
			Heart failure	RRR 0.65
			Cerebrovascular disease	RRR 11.55
			Chronic obstructive pulmonary disease	RRR 0.73
			Alcoholic liver disease	RRR 4.03
			Coxarthrosis	RRR 4.93
			Gonarthrosis	RRR 3.89
			Glomerular disorders	RRR 0.32
			Intracranial injury	RRR 2.21
			Fracture of elbow and forearm	RRR 2.41
			Fracture of femur	RRR 9.30
			Fracture of ankle/lower leg	RRR 8.18
		Length of admission (days)		RRR 1.12
		Presence of spouse		RRR 0.48
		Presence of child		RRR 1.17
Zureik [43]	Age; living alone; patient wishes; family wishes; cognitive impairment; functional ability; co-morbidities; admission diagnosis; prior care	Age (>85 versus ≤85)		OR 1.8 (1.1–2.9)
		Living alone		OR 1.9 (1.2–3.3)
		Family wishes No opinion/no carer		OR 2.9 (1.9–4.3)
		Opposition to going home		OR 8.2 (3.5–18.9)
		Mild ‘mental alteration’		OR 1.4 (1.0–1.7)
		Moderate ‘mental alteration’		OR 1.8 (1.1–2.8)
		Severe ‘mental alteration’		OR 2.3 (1.3–4.8)
		Functional ability (ADL score on admission) 1–3		OR 1.5 (1.0–2.0)
		4–6		OR 2.1 (1.5–3.9)
		Co-morbidities (chronic conditions; degree of fatality)		
		Non-fatal		OR 2.1 (1.3–3.3)
		Fatal		OR 4.3 (1.7–10.7)

*Notes*: BADLs, basic activities of daily living; RRR, Relative Risk Ratio; CAM, confusion assessment method; C-MMSE, Chinese Mini Mental State Examination; D, development cohort; IADLs, instrumental activities of daily living; V, validation cohort. *Data reported on significant predictors for nursing home admission, versus home with home care and home for the elderly care. Reported in text as relative risk ratios with standard error and annotation to denote statistical significance.

#### Meta-analyses

Including data from 11 studies which reported either multivariate models or adjusted analyses of a single predictor, we calculated summary estimates for five predictors: age, female sex, delirium, dementia and cognitive impairment and functional dependency (Table 3; Supplementary data, Figure 2, are available in *Age and Ageing* online). Care home admission was associated with increased age (per year increase) (Pooled OR 1.02 95% CI: 1.00–1.04; 4,431 participants; five studies; moderate quality evidence); female sex (Pooled OR 1.41 95% CI: 1.03–1.92; 8,312 participants; five studies; low-quality evidence); dementia and cognitive impairment (Pooled OR 2.14 95% CI: 1.24–3.70; 4,018 participants; five studies; low-quality evidence); functional dependency (Pooled OR 2.06 95% CI: 1.58–2.69; 7,796 participants; six studies; moderate quality evidence). Delirium was not associated with care home admission (Pooled OR 1.61 95% CI: 0.82–3.17; 3,267 participants; three studies; very low-quality evidence).

**Table 3.** Results of quantitative synthesis

Predictors	Number of studies	Number of participants	Pooled OR (95% CI)	*I*^2^ % (Statistical heterogeneity)	GRADE assessment	Rationale
Age	5 studies 6 data sets*	4,431	1.02 (1.00–1.04)	0	⊕⊕⊕⊖ Moderate quality evidence	Downgraded due to risks of selection bias and reporting bias
Female sex	5 studies	8,312	1.41 (1.03–1.92)	15	⊕⊕⊖⊖ Low-quality evidence	Downgraded due to risks of selection bias and reporting bias and inconsistency
Dementia and cognitive impairment	5 studies 6 data sets*	4,018	2.14 (1.24–3.70)	2	⊕⊕⊖⊖ Low-quality evidence	Downgraded due to risks of selection bias and reporting bias and inconsistency
Delirium	3 studies 4 data sets*	3,267	1.61 (0.82–3.17)	20	⊕⊖⊖⊖ Very low-quality evidence	Downgraded due to risks of selection bias, inconsistency and imprecision
Functional dependency	6 studies	7,796	2.06 (1.58–2.69)	0	⊕⊕⊕⊖ Moderate quality evidence	Downgraded due to risks of selection bias and reporting bias

*Data from development and validation cohorts within the same study.

#### Studies using other designs

Three studies, not included in the existing analyses, examined the effects of a single factor on the outcome of care home admission, adjusted for other potential confounders. Bonneyfoy *et al.* determined that malnutrition was associated with care home admission RR 2.04 (1.23–3.38; 1066 participants) [27]. Gordon *et al.* identified that being unmarried was associated with an increased risk of care home admission, OR 2.67 (2.22–3.06; 40,820 participants) [32]. Romero-Ortuno's Risk Index for Geriatric Acute Medical Admissions, adjusted for age, was associated with care home admission for those with 3–5 deficits OR 1.34 (1.05–1.72; 15,873 participants) [39].

Brown *et al.* evaluated a series of routinely collected biochemical test results on the likelihood of care home admission, adjusted for likely confounding factors, with a sample of 392 participants [29]. None of the observed associations between abnormal test results and care home admission persisted after adjustment [29].

Adamis *et al. *conducted binary logistic regression analysis, but presented Wald statistics results rather than ratios, identifying age (years) 8.39 (*P* = 0.004) and delirium 7.04 (*P* = 0.008) as being associated with care home admission in 94 participants [22].

Astell *et al.* examined the correlation between predictors and discharge to home, nursing home care and in-hospital death in 234 participants, using multinomial logistic regression. This study concluded that dependency (Spearman's rank-order correlation *r*s −0.274) and active medical problem count (rs −0.336) were negatively correlated with nursing home admission suggesting lower dependency predicted admission to nursing home. Correspondence with the author has clarified that this is because those with greatest dependency and medical problems were most likely to die in hospital (survival bias) [24].

Two studies presented unadjusted analyses which only examined a single predictor [31, 41]. Malnutrition was associated with higher risk of discharge to a nursing home (malnourished 20.3% versus not 7.7%, *P* < 0.001; 1,145 participants) [41]. Experiencing a fall at any point during admission was associated with nursing home placement (any fall 12.9% versus no fall 5.6%; *P* < 0.005; 620 participants) [31].

### Sub-group analyses

*Residential versus nursing care*: Only one study (Wong *et al.* 262,439 participants) evaluated predictors for two levels of institutional care, defined as nursing homes or homes for the elderly in the Netherlands [42]. Two factors were associated with home for the elderly care but not nursing home care: female sex and the presence of female spouse [42]. Five diagnoses were associated with nursing home care but not home for the elderly care: bladder cancer, Alzheimer's disease, heart failure, chronic obstructive pulmonary disease, bladder cancer and glomerular disorders [42]. Otherwise, all other variables which were associated with nursing home care (Table 2) were also associated with home for the elderly care [42].

*Country of origin*: No trends were noted by country of origin. In view of the heterogeneity of the data and difficulties in pooling results this has not been formally evaluated.

*Age <65 versus ≥65*: Only one study had a mean age <65 years and so sub-group analysis was not possible.

*Timing of assessment of predictor*: Luk *et al.* evaluated predictors of care home admission at time of hospital admission and at discharge in 535 participants [37]. They report results of functional assessment, mobility, cognition, albumin, incontinence, catheterisation and pressure sores with statistically significant proportional differences at each stage between those who are discharged home and those admitted to a care home [37].

*Dementia and delirium*: Three studies evaluated the role of diagnosed dementia as a predictor of care home admission in 265,149 participants [25, 30, 42]. In two of these there was evidence of a statistically significant positive association between dementia and care home admission [25, 42]. Five studies evaluated delirium as a predictor of care home admission in 3,958 participants [22, 25, 28, 30, 33]. There was evidence of a statistically significant positive association between delirium and care home admission identified in three studies, although the included populations and methods of analysis were heterogeneous [22, 28, 33].

## Discussion

### Summary of evidence

This review included a study population of 354,985 participants admitted directly to long-term institutional care from the acute hospital, from 12 countries and 23 studies. There was significant variation in the likelihood of new care home admission at hospital discharge. Despite the size of the available evidence, heterogeneity and poor quality of reporting mean that we cannot determine all the predictors of care home admission following acute hospitalisation. The presentation of the results is largely narrative, with only 11 studies contributing to quantitative synthesis. Where we were able to offer summary estimates the supporting evidence is of moderate to low quality. In keeping with clinical experience, identified significant predictors include age, female sex, dementia and increasing functional dependency. Surprisingly, social support, family and patient wishes, the availability and costs of social care and other clinical variables such as continence and BPSD were rarely or not reported. None of the included studies examined in-hospital care processes or adverse events.

### Comparison with predictors from community-based cohorts

Increased age has been identified as a predictor of care home admission in community cohorts [7] and specifically among those with dementia [44]. However, sex as a predictor variable has produced inconsistent results previously [7], and caregiving status and support was not explored in our included studies. Dementia and cognitive impairment are key predictors of care home admission [45, 46], with severity of dementia increasing risk [47]. Our review was limited by the varied measures used to evaluate dementia and cognitive impairment across the included studies. Although cognitive tests scores were considered, no formal measure of disease severity was used. Such heterogeneity in measurement has been observed before [48, 49] and is a limitation in this field. No studies looked at BPSD as a predictor to allow comparison with findings seen in non-hospitalised cohorts [9].

The review data regarding delirium are difficult to pool, due to the varied methods applied in the included studies. A previous systematic review looking at delirium outcomes found a strong association with care-home admission in medium to longer term follow-up [50]. Our data did not suggest that delirium was associated with immediate institutionalisation. This is an intriguing result and suggests that the adverse consequences of a delirium episode continue beyond the acute admission.

Dependence in ADL has also been established as a predictor of care home admission among inpatients [51] and in those with dementia in community settings [47, 52]. Living alone, widowhood and caregiver burden are factors which have been considered in greater detail in non-hospitalised cohorts [44, 53, 54]. Emotional factors such as loneliness, which is known to be associated with requiring institutional care [55, 56], were not evaluated. Qualitative interview data from carers for people with dementia have indicated greater complexity in reasons for institutionalisation, related to culture, organisation of services and relationships [57]. These are all potentially important explanatory variables which may not be recorded in routine healthcare data. Luppa *et al.* [52] present a conceptual framework to analyse factors influencing institutionalisation for adults with dementia, acknowledging the interacting roles of predisposing (sociodemographic and relationship characteristics), need (stressors) and enabling variables (resources). Greater understanding of how these factors impact on decisions made in hospital would be valuable.

### Strengths and limitations

This review's protocol was registered, and it was reported in accordance with the PRISMA guidelines [58]. The search strategy was comprehensive and inclusive. Eligibility was determined by the evaluation of two independent reviewers. The risk of bias assessment was based on an established tool, RoBANS [18], modelled on the Cochrane Risk of Bias assessment [58]. Quantitative synthesis has been performed where data allowed and the quality of evidence has been evaluated using a recognised system, GRADE [20].

Selection bias affected the majority of included studies. Methodological quality has been identified as an area requiring improvement for community and dementia-specific studies of predictors of care home placement [7, 52].

Only one study had a published protocol to allow readers to evaluate the risk of reporting bias. Registration of clinical trials and publication of protocols has helped to ensure greater transparency and evaluation of findings [60]. Although these have been less common in observational research [60], greater use of protocols could lead to similar improvements and is to be encouraged.

With a median sample size of 727 participants and much larger data sets contributing, evaluation of statistically significant predictors was limited not by size, but by comparability of measurement techniques. For three of the five statistical comparisons, data originated from two cohorts within the same study and so are not independent. Failure to evaluate possible confounding variables  was encountered in studies which presented unadjusted analyses, but also among those who did not evaluate the role of cognition, age, sex, comorbidity or function in these typically older cohorts. Future research would benefit from the use of the Strengthening the Reporting of Observational Studies in Epidemiology (STROBE) guideline to ensure more complete reporting [62].

The scope of the factors evaluated by the included studies were limited. Furthermore, patient wishes and family wishes were only evaluated in a single study and other variables such as level of social care, financial support and caregiver stress were not considered. This failure to evaluate the wider individual and organisational drivers of this life-changing decision is a significant limitation and this topic requires mixed methods research.

None of the studies described the nature of provision in the care home setting and this limits the ability of readers to determine if the study is comparable to their practice. Although this review sought studies where the care home was the new place of permanent residency for the individual, care homes are increasingly being used for the provision of rehabilitation and other forms of post-acute care. It is possible some of the studies included individuals moving to this setting for temporary care and the strength of association with dependency may be compounded by this. The need for authors to provide a clear description of care model has been advocated by the international care home research community [15].

## Conclusions

### Implications for practice

Discharge to long-term institutional care following acute hospital admission is a common outcome with significant variation (3–77% (median 15%)), however, current data do not allow us to predict who will make this transition. Older, female individuals with dementia and functional dependency are more likely to be admitted to institutional long-term care. These variables are clinically valid, but lack discriminatory power in the acute hospital.

### Implications for research

We recommend that future research includes: publication of study protocols; standardisation of analysis methods and study reporting; reporting of all results irrespective of statistical significance; greater inclusiveness in study design to reduce selection bias; more consideration of likely confounding variables in this population—especially dementia and delirium; and clear descriptions of the model of care provided in the care home and hospital. It is imperative that any further research measures variables which are most relevant to the individuals who experience this transition and this may require bespoke data collection to capture these complimented by qualitative research to ensure patient perspectives are explored.

**Key points**

- Institutional long-term care (‘care home’) admission following acute hospitalisation is common.
- Older age, female sex, dementia and functional dependence are established predictors.
- Social support, patient preferences and other potential contributory factors have not been adequately evaluated in hospitalised cohorts.
- Further research is required to better understand predictors of care home admission from the acute hospital.
- Greater clarity is needed from study authors on the nature of care provided in ‘care home’ settings.

## Supplementary Material

Supplementary Data
