# Supplementary material for: Predicting discharge to institutional long-term care following acute hospitalisation: a systematic review and meta-analysis
Source: Age Ageing. 2017 Jun 20;46(4):547–58. doi: 10.1093/ageing/afx047 (PMC5860007; doi:10.1093/ageing/afx047)
Supplement: Supplementary Data [file supplementary_data.docx]

**Predicting discharge to institutional long-term care from the acute hospital setting: a systematic review & meta-analysis**

**Supplementary Data**

For Age and Ageing paper: *Predicting discharge to institutional long-term care from the acute hospital setting: a systematic review & meta-analysis*

**Supplementary Text: Data items**

**Supplementary Figure 1: Risk of Bias Summary Chart**

**Supplementary Table 1: Included study population characteristics**

Supplementary Figure 2: Forest plots from quantitative analyses

**Appendix 1: Search Strategy**

**Appendix 2: Quality Assessment Criteria**

**Appendix 3: full list of references**

**Supplementary text:**

**Data items**

Data were extracted on the following items: sample size, country, study design, prospective/retrospective, data collection period, setting, disease/condition-specific, age (with statistics as reported), proportion male sex, proportion of those surviving to discharge to who were newly discharged to a care home, type of care home, definition of care home, dementia diagnosis and other comorbidities and the purpose of the original study was recorded. Data were extracted to facilitate risk of bias assessment (discussed below). Possible predictors included: age, sex, living alone, patient wishes, family wishes, cognitive impairment, dementia, delirium, nutrition, mobility, functional ability, continence, polypharmacy, admission diagnosis, co-morbidities, length of admission, recurrent previous admissions, prior care and end of life care. Additional free space was included to record other predictive variables and any other relevant data identified by the assessor.


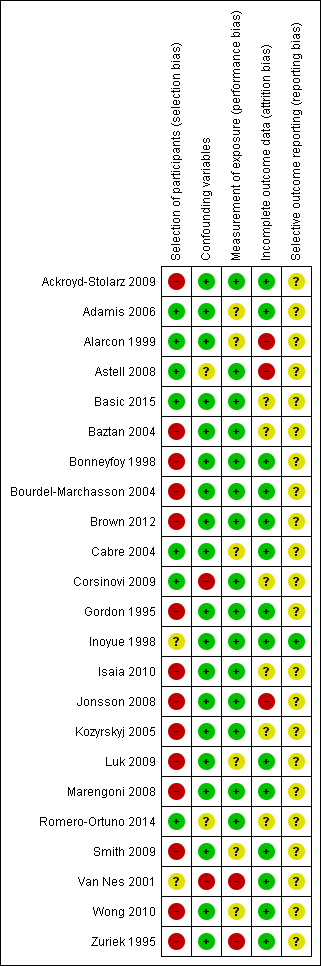
**Supplementary Figure 1: Risk of Bias Summary Chart**

Supplementary Table 1: Included study population characteristics

| **Study ID/Year** | **Mean Age (SD)** | **Male sex %** | **Inpatient death (%)** | **Discharged to CH (%)** | **Dementia diagnosis** | **Other comorbidities** |
| --- | --- | --- | --- | --- | --- | --- |
| Ackroyd-Stolarz 2009 | 78 (8) | 48% | 136 (14%) | 29 (3%) | Not reported | Not reported |
| Adamis 2006 | 83.8 (6.5) | 40% | 9 (10%) | 17 (20%) | Not reported | Not reported |
| Alarcon 1999 | 81.8 (7.2) | 34% | 37 (11) | 10 (3%) | Not reported | Not reported |
| Astell 2008 | Not reported | 44% | 55 (24%) | 137 (77%) | Using MMSE  38% severe;  41.5% moderate; 20.5% mild | Number of physical complaints reported; 12% had >6 |
| Basic 2015 | Dev 83.0 (7.9)  Val 82.7 (7.5) | D 41.8%;  Val 40% | Dev 9.6% Val 10.4% | Overall 6.4% (unable to say if accounts for deaths as no figures reported) | Dev 40.9%;  Val 43.7% | Data on delirium, BPSD, acute renal failure and other comorbidities |
| Baztan 2004 | 80.56 (7.45) | 35% | Not reported | 16% | 27% scored ≥5 on SPMSQ | Data reported in Charlson Index at baseline |
| Bonneyfoy 1998 | 82.7 (6.6) | 31% | 84 (7.9%) | 595 (61%) | 53.4% ‘Abnormal mental status’ | Defined by ICD-9 characteristics |
| Bourdel-Marchasson 2004 | Institution:  85.6 (6.8); Community:  84.6 (6.2) | Institution: 26%  Community: 52% | Excluded | 117 (27%) | Cog Imp:  48% community;  61% institution | Charlson index reported for each group |
| Brown 2012 | 83.2 (5.5) | 72% | 33 (8%) | 38 (11%) | AMT mean score 8.1 (3.2) | Mean number 3.8 (1.8) |
| Cabre 2004 | 84 (6.2) | 46% | 26 (4%) | 90 (16%) | 14.7% of those who went home; 30% of those institutionalised | Data reported on proportion in each group with categories of comorbidities |
| Corsinovi 2009 | Not reported | Not reported | Not reported | Not reported | Not reported | Not reported |
| Gordon 1995 | Unmarried 55 (22);  Married 58 (16) | 46% | 1647 (4%) | 1631 (4%) | Not reported | Admission severity of illness score |

| **Study ID/Year** | **Mean Age (SD)** | **Male sex %** | **Inpatient death (%)** | **Discharged to CH (%)** | **Dementia diagnosis** | **Other comorbidities** |
| --- | --- | --- | --- | --- | --- | --- |
| Inouye 1998 | 78.9 (6.9) | 40% | 35 (5%) | 60 (9%) | 19% | Data reported on primary medical problem |
| Isaia 2010 | 82.2 (7.3) | 33% | 14 (5%) | Not reported | Mean MMSE 23.9 (8.1) | Data on APACHE II and GDS reported at baseline |
| Jonsson 2008 | 83.7 (5.4) | 34% | 42 (6%) | 81 (12%) | Mod/severe cognitive decline 18% | Data on number of diagnoses reported |
| Kozyrskyj 2005 | Not reported | Not reported | (22%) | 19% | Not reported | Not reported |
| Luk 2009 | 80.6 (6.89) | 43% | Reported as composite death/transfer | 116 (22%) | Mean MMSE 17.1 (6.3) | Not reported |
| Marengoni 2008 | 78.5 (7.2) | 50% | 64 (7%) | 23 (3%) | 39% cognitive impairment (MMSE <24) | Data on diseases and disorders on admission reported |
| Romero-Ortuno 2014 | ND 76.3 (7.3);  1-2D 77.4 (7.5);  3-5D 79.3 (7.6);  ≥6D 80.9 (7.6) | Not reported | Not reported | Not reported | Not reported | Not reported |
| Smith 2009 | Not reported | 50% | Not reported | 981 (16%) | Not reported | Not reported |
| Van Nes 2001 | 84.1 (6.7) | 30% | 80 (7%) | 147 (13%) | Not reported | Not reported |
| Wong 2010 | 74.2 (6.4) | 50% | Not reported | 9049 (3%) | 0.40% | Data on baseline comorbidities reported |
| Zureik 1995 | 84.3 (5.5) | 27% | 71 (14%) | 169 (41%) | ‘Mental alteration 54%' | Data on acute and chronic conditions at baseline reported |

*Notes: APACHE II – Acute Physiology and Chronic Health Evaluation; Cog Imp – cognitive impairment; D – deficits; Dev – development cohort; GDS – Geriatric Depression Scale; ICD-9 – international classification of diseases version 9; MMSE – mini mental state examination; ND – no deficits; SPMSQ -* Short Portable Mental Status Questionnaire; *Val – validation cohort.*

*All percentages rounded to nearest whole number*

Supplementary Figure 2: Forest plots from quantitative analyses


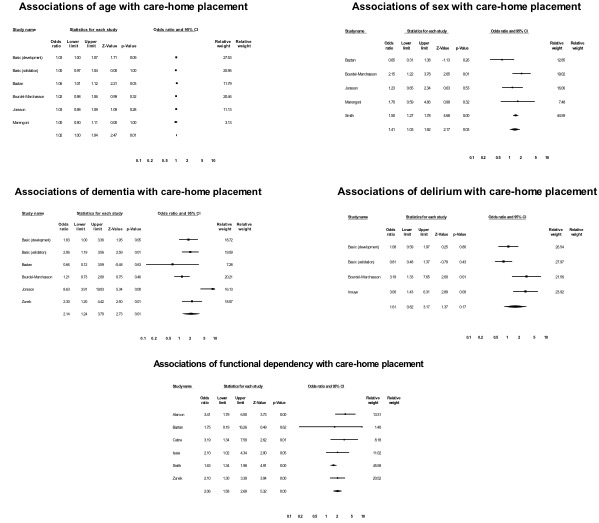


**Appendix 1: Search Strategy**

*The final search for each database combined: Concepts 1, 2 & 3 with AND*

Ovid MEDLINE (R) In Process and Other Non-Indexed Citations and Ovid MEDLINE (R) 1946 to present

Search Concept 1: Care home

nursing home.mp.2. OR (care home or care home*).mp. OR Residential Facilities/ or residential.mp. OR institutionalisation.mp. OR institutionalization.mp. or exp Institutionalization/ OR "long term care".mp. or Long-Term Care/ OR exp Homes for the Aged/ OR ((aged or elderly) adj2 (care or facilit* or home*)).ti,ab.

Search Concept 2: Hospitalisation

Hospitalisation: hospital.mp. or Hospitals/ OR inpatient.mp. or Inpatients/ OR in-patient.mp. OR hospitalisation.mp. OR Hospitalization/ or hospitalization.mp. OR hospitalised.mp.

Search Concept 3: Risk/predictive factor

Risk/predictive factor: risk factor.mp. or exp Risk Factors/ OR cause.mp. OR causes.mp. OR predict.mp. OR predictor.mp.

Ovid EMBASE 1980 to 2015 Week 39

Search Concept 1: Care home

nursing home.mp. or nursing home/ OR care home.mp. OR care home*.mp. OR residential care/ or residential.mp. or residential home/ OR institutionalisation.mp. OR institutionalization.mp. or exp institutionalization/ OR long term care.mp. or long term care/ OR exp home for the aged/ OR ((aged or elderly) adj2 (care or facilit* or home*)).ti,ab.

Search Concept 2: Hospitalisation

hospital/ or hospital.mp. OR inpatient.mp. OR in-patient.mp. OR hospitalisation.mp. OR hospitalization/ or hospitalization.mp. OR hospitalised.mp.

Search Concept 3: Risk/predictive factor

risk factor.mp. or exp risk factor/ OR cause.mp. OR causes.mp. OR predict.mp. OR predictor.mp.

EBSCOhost CINAHL Plus

Search Concept 1: Care home

(MH “Nursing Homes”) OR “nursing home” OR “care home*” OR “care home” OR (MH “Residential Care”) OR “residential” OR (MH “Residential Facilities”) OR “institutionalisation” OR (MH “Institutionalization”) OR “institutionalization” OR (MH “Long Term Care”) OR “long term care”

Search Concept 2: Hospitalisation

“hospital” OR (MH “Hospitals”) OR “inpatient” OR (MH “Inpatients”) OR “in-patient” OR “hospitalisation” OR (MH “Hospitalization”) OR “hospitalization” OR “hospitalised”

Search Concept 3: Risk/predictive factor

(MH “Risk Factors+”) OR “risk factor” OR “cause” OR “causes” OR “predict” OR “predictor”

**Appendix 2: Quality Assessment Criteria**

| **Risk of Bias Item** | **Grading** | **Rationale/Examples** |
| --- | --- | --- |
| **Selection of participants** *(selection bias)* | Low | Sampling frame clearly described with reasonable inclusion/exclusion criteria provided, ideally allowing for inclusion of all participants admitted to the hospital (or specialist service)  Exclusion of those admitted from nursing/residential care and those who did not survive to discharge is considered appropriate |
|  | High | Exclusions based on availability of data (e.g. transfer to other ward/hospital/department; admission to ITU); studies only considering first admission or excluding those with prior admissions/attendances at hospital; exclusion criteria likely to reduce the representativeness of a possible care home population (e.g. comorbidities, end of life care etc.); exclusion of those lost to follow-up; non-consecutive samples if consecutive sample sought |
|  | Unclear | Sampling frame unclear, criteria for inclusion/exclusion not provided or explained |
| **Confounding variables** | Low | Multivariate model accounting for likely possible confounding variables |
|  | High | No consideration of confounding variables; univariate analyses only |
|  | Unclear | Methods for analysis not clearly described or reported |
| **Measurement of exposure** *(performance bias)* | Low | Clearly described method on how data were collected and extracted  Best practice includes description of who performed data extraction, case definitions/descriptions of eligible conditions |
|  | High | Missing data on key predictor variables |
|  | Unclear | Methods for assessing predictor variables not clearly described |
| **Incomplete outcome data**  *(attrition bias)* | Low | Outcomes assessed for all included participants |
|  | High | Missing outcome assessments |
|  | Unclear | Outcome assessment reported as percentages without absolute values being presented, preventing assessment of completeness of outcome reporting |
| **Selective outcome reporting**  *(reporting bias)* | Low | Reporting as per published protocol |
|  | High | Evidence that reporting deviates from publically accessible protocol |
|  | Unclear | No protocol publically available |

**Appendix. Full list of References**

1. Ribbe MW, Ljunggren G, Steel K, Topinkova E, Hawes C, Ikegami N, et al. Nursing homes in 10 nations: a comparison between countries and settings. Age and Ageing. 1997 Sep;26 Suppl 2:3-12.

2. Wubker A, Zwakhalen SM, Challis D, Suhonen R, Karlsson S, Zabalegui A, et al. Costs of care for people with dementia just before and after nursing home placement: primary data from eight European countries. The European journal of health economics : HEPAC : health economics in prevention and care. 2015 Sep;16(7):689-707.

3. Audit Scotland. Reshaping care for older people2014 23rd November 2015 [cited 2015 23rd November]: Available from: <http://www.audit-scotland.gov.uk/docs/central/2014/nr_140206_reshaping_care.pdf>.

4. NHS England. Safe, compassionate care for frail older people using an integrated care pathway: Practical guidance for commissioners, providers and nursing, medical and allied health professional leaders2014 16th October 2015: Available from: <https://www.england.nhs.uk/wp-content/uploads/2014/02/safe-comp-care.pdf>.

5. Joint Improvement Team. Home First - Ten Actions to Transform Discharge. Scotland: 2015.

6. Alzheimer's Society. Support. Stay. Save. Care and support of people with dementia in their own homes 2011 29th September 2015 [cited 2015 29th September]: Available from: <http://www.alzheimers.org.uk/site/scripts/download_info.php?downloadID=526>.

7. Luppa M, Luck T, Weyerer S, Konig HH, Brahler E, Riedel-Heller SG. Prediction of institutionalization in the elderly. A systematic review. Age and Ageing. 2010;39(1):31-8.

8. Verbeek H, Meyer G, Challis D, Zabalegui A, Soto ME, Saks K, et al. Inter-country exploration of factors associated with admission to long-term institutional dementia care: evidence from the RightTimePlaceCare study. J Adv Nurs. 2015 Jun;71(6):1338-50.

9. Toot S, Swinson T, Devine M, Challis D, Orrell M. Causes of nursing home placement for older people with dementia: a systematic review and meta-analysis. International Psychogeriatrics. 2016:1-14.

10. Imison C, Poteliakhoff E, Thompson J. Older people and emergency bed use - Exploring variations2012: Available from: <http://www.kingsfund.org.uk/sites/files/kf/field/field_publication_file/older-people-and-emergency-bed-use-aug-2012.pdf>.

11. Oliver D, Foot C, Humphries R. Making our health and care systems fit for an ageing population2014 10th December 2015: Available from: <http://www.kingsfund.org.uk/sites/files/kf/field/field_publication_file/making-health-care-systems-fit-ageing-population-oliver-foot-humphries-mar14.pdf>.

12. Nardi R, Scanelli G, Tragnone A, Lolli A, Kalfus P, Baldini A, et al. Difficult hospital discharges in internal medicine wards. Internal and emergency medicine. 2007 Jun;2(2):95-9.

13. Ellis G, Whitehead M, O'Neill D, Langhorne P, Robinson D. Comprehensive geriatric assessment for older adults admitted to hospital. Cochrane Database of Systematic Reviews. 2011(7).

14. Liberati A, Altman D, Tetzlaff J, Mulrow C, Gotzsche P, Ioannidis J, et al. The PRISMA Statement for Reporting Systematic Reviews and Meta-Analyses of Studies That Evaluate Health Care Interventions: Explanation and Elaboration. PLoS Med. 2009;6(7):e10000100.

15. Sanford AM, Orrell M, Tolson D, Abbatecola AM, Arai H, Bauer JM, et al. An international definition for "nursing home". J Am Med Dir Assoc. 2015 Mar;16(3):181-4.

16. Armstrong C, Hamilton L, Shenkin S. Factors Predictive of Nursing Home Admission Directly From Hospital: A Systematic Review. [Personal communication]. Personal communication ed2014.

17. Covidence. Covidence lets you create and maintain Systematic Reviews online. Melbourne, Australia: Alfred Health; 2015; Available from: [www.covidence.org](http://www.covidence.org)

18. Kim S, Park J, Lee Y, Seo H-J, Sheen S-S, Hahn S, et al. Testing a tool for assessing the risk of bias for nonrandomized studies showed moderate reliability and promising validity. Journal of Clinical Epidemiology. 2013;66:408-14.

19. Borenstein M, Hedges L, Higgins J, Rothstein H. Comprehensive Meta Analysis. 3.0 ed. USA: Biostat; 2004.

20. Guyatt GH, Oxman AD, Vist GE, Kunz R, Falck-Ytter Y, Alonso-Coello P, et al. GRADE: an emerging consensus on rating quality of evidence and strength of recommendations. BMJ. 2008;336(7650):924-6.

21. Ackroyd-Stolarz S, Guernsey JR, MacKinnon NJ, Kovacs G. Impact of adverse events on hospital disposition in community-dwelling seniors admitted to acute care. Healthcare Quarterly. 2009;12:34-9.

22. Adamis D, Treloar A, Martin F, Macdonald A. Recovery and outcome of delirium in elderly medical inpatients. Archives of Gerontology and Geriatrics. 2006;43:289-98.

23. Alarcon T, Barcena A, Gonzalez-Montalvo J, Penalosa C, Salgado A. Factors predictive of outcome on admission to an acute geriatric ward. Age and Ageing. 1999;28:429-32.

24. Astell AJ, Clark SA, Hartley NT. Predictors of discharge destination for 234 patients admitted to a combined geriatric medicine/old age psychiatry unit. International journal of geriatric psychiatry. 2008 Sep;23(9):903-8.

25. Basic D, Shanley C. Frailty in an Older Inpatient Population: Using the Clinical Frailty Scale to Predict Patient Outcomes. Journal of Aging & Health. 2015;27(4):670-85.

26. Baztan JJ, Gonzalez M, Morales C, Vazquez E, Moron N, Forcano S, et al. [Variables associated with functional recovery and post-discharge institutionalization of elderly cared in an average stay geriatric unit]. Revista clinica espanola. 2004 Nov;204(11):574-82.

27. Bonnefoy M, Ayzac L, Ingenbleek Y, Kostka T, Boisson RC, Bienvenu J. Usefulness of the prognostic inflammatory and nutritional index (PINI) in hospitalized elderly patients. International journal for vitamin and nutrition research. 1998;68(3):189-95.

28. Bourdel-Marchasson I, Vincent S, Germain C, Salles N, Jenn J, Rasoamanarivo E, et al. Delirium Symptoms and Low Dietary Intake in Older Inpatients Are Independent Predictors of Institutionalization: A 1-Year Prospective Population-Based Study. Journal of Gerontology: Medical Sciences. 2004;59A(4):350-4.

29. Brown SH, Flint K, Storey A, Abdelhafiz AH. Routinely assessed biochemical markers tested on admission as predictors of adverse outcomes in hospitalized elderly patients. Hospital practice (1995). 2012 Feb;40(1):193-201.

30. Cabre M, Serra-Prat M. Determinants of admission to nursing homes in elderly inpatients. Revista Espanola de Geriatria y Gerontologia. 2004;39(6):367-70.

31. Corsinovi L, Bo M, Ricauda Aimonino N, Marinello R, Gariglio F, Marchetto C, et al. Predictors of falls and hospitalization outcomes in elderly patients admitted to an acute geriatric unit. Archives of Gerontology and Geriatrics. 2009 Jul-Aug;49(1):142-5.

32. Gordon HS, Rosenthal GE. Impact of marital status on outcomes in hospitalized patients. Evidence from an academic medical center. Archives of Internal Medicine. 1995 Dec 11-25;155(22):2465-71.

33. Inouye S, Rushing J, Foreman M, Palmer R, Pompei P. Does Delirium Contribute to Poor Hospital Outcomes? A Three-Site Epidemiologic Study. Journal of General Internal Medicine. 1998;13:234-42.

34. Isaia G, Bo M, Aimonino N, Isaia G, Michelis G, Miceli C, et al. Functional decline two weeks before hospitalization in an elderly population. Aging Clinical & Experimental Research. 2010;22(4):352-5.

35. Jonsson P, Noro A, Finne-Soveri H, Jensdottir A, Ljunggren G, Bucht G, et al. Admission profile is predictive of outcome in acute hospital care. Aging Clinical and Experimental Research. 2008;20:533-9.

36. Kozyrskyi AL, Black C, Chateau D, Steinbach C. Discharge outcomes in seniors hospitalized for more than 30 days. Canadian Journal on Aging. 2005 Spring;24 Suppl 1:107-19.

37. Luk J, Chiu P, Chu L. Factors affecting institutionalization in older Hong Kong Chinese patients after recovery from acute medical illnesses. Archives of Gerontology and Geriatrics. 2009;49:e110-e4.

38. Marengoni A, Aguero-Torres H, Timpini A, Cossi S, Fratiglioni L. Rehabilitation and Nursing Home Admission after Hospitalization in Acute Geriatric Patients. Journal of the American Medical Directors Association. 2008;9:265-70.

39. Romero-Ortuno R, O'Dwyer C, Byrne D, O'Riordan D, Silke B. A Risk Index for Geriatric Acute Medical Admissions (RIGAMA). Acute medicine. 2014;13(1):6-11.

40. Smith E, Stevens A. Predictors of Discharges to a Nursing Home in a Hospital-Based Cohort. Journal of the American Medical Directors Association. 2009;10:623-9.

41. Van Nes M-C, Herrmann F, Gold G, Michel J-P, Rizzoli R. Does the Mini Nutritional Assessment predict hospitalization outcomes in older people? Age and Ageing. 2001;30:221-6.

42. Wong A, Elderkamp-de Groot R, Polder J, van Exel J. Predictors of Long-Term Care Utilization by Dutch Hospital Patients aged 65+. BMC Health Services Research. 2010;10:110.

43. Zureik M, Lang T, Trouilet J-L, Davido A, Tran B, Levy A, et al. Returning Home after Acute Hospitalization in Two French Teaching Hospitals: Predictive Value of Patients' and Relatives' Wishes. Age and Ageing. 1995;1995(24).

44. Eska K, Graessel E, Donath C, Schwarzkopf L, Lauterberg J, Holle R. Predictors of Institutionalization of Dementia Patients in Mild and Moderate Stages: A 4-Year Prospective Analysis. Dementia and Geriatric Cognitive Disorders Extra. 2013;3:426-45.

45. Zekry D, Herrmann F, Grandjean R, Vitale A-M, De Pinho M-F, Michel J-P, et al. Does dementia predict adverse hospitalization outcomes? A prospective study in aged inpatients. International Journal of Geriatric Psychiatry. 2009;24:283-91.

46. Hajek A, Brettschneider C, Lange C, Posselt T, Wiese B, Steinmann S, et al. Longitudinal Predictors of Institutionalization in Old Age. PLoS One. 2015;10(12):e0144203.

47. Gaugler J, Yu F, Krichbaum K, Wyman J. Predictors of Nursing Home Admission for Persons with Dementia. Medical Care. 2009;47(2):191-8.

48. Lees R, Fearon P, Harrison J, Broomfield N, Quinn T. Cognitive and mood assessment in stroke research: focused review of contemporary studies. Stroke. 2012;43(6):1678-80.

49. Harrison J, Noel-Storr A, Demeyere N, Reynish E, Quinn T. Outcome Measures in a Decade of Dementia and Mild Cognitive Impairment Trials. Alzheimer's Research & Therapy. 2016;8:48.

50. De Buyser SL, Petrovic M, Taes YE, Vetrano DL, Onder G. A multicomponent approach to identify predictors of hospital outcomes in older in-patients: a multicentre, observational study. PLoS One. 2014;9(12):e115413.

51. Luppa M, Luck T, Brahler E, Konig H-H, Riedel-Heller SG. Prediction of Institutionalisation in Dementia. A systematic review. Dementia and Geriatric Cognitive Disorders. 2008;26(1):65-78.

52. Verbeek H, Meyer G, Leino-Kilpi H, Zabalegui A, Hallberg I, Saks K, et al. A European study investigating patterns of transition from home care towards institutional dementia care: the protocol of a RightTimePlaceCare study. BMC Public Health. 2012;12(1):68.

53. McCann M, Donnelly M, O'Reilly D. Living arrangements, relationship to people in the household and admission to care homes for older people. Age and Ageing. 2011 May;40(3):358-63.

54. Tijhuis MA, De Jong-Gierveld J, Feskens EJ, Kromhout D. Changes in and factors related to loneliness in older men. The Zutphen Elderly Study. Age Ageing. 1999 Sep;28(5):491-5.

55. Tilvis RS, Pitkala KH, Jolkkonen J, Strandberg TE. Social networks and dementia. Lancet (London, England). 2000 Jul 01;356(9223):77-8.

56. Afram B, Stephan A, Verbeek H, Bleijlevens MHC, Suhonen R, Sutcliffe C, et al. Reasons for Institutionalization of People With Dementia: Informal Caregiver Reports From 8 European Countries. Journal of the American Medical Directors Association. 2014;15(2):108-16.

57. Moher D, Liberati A, Tetzlaff J, DG A, Group TP. Preferred Reporting Items for Systematic Reviews and Meta-Analyses: The PRISMA Statement. PLoS Med. 2009;6(6):e1000097.

58. Higgins JPT, Altman DG, Gøtzsche PC, Jüni P, Moher D, Oxman AD, et al. The Cochrane Collaboration’s tool for assessing risk of bias in randomised trials. BMJ. 2011;343:d5928.

59. Dwan K, Altman DG, Cresswell L, Blundell M, Gamble C, Williamson P. Comparison of protocols and registry entires to published reports for randomised controlled trials. Cochrane Database of Systematic Reviews. 2011(1):Art. No.: MR000031.

60. Norris SL, Moher D, Reeves BC, Shea B, Loke Y, Garner S, et al. Issues relating to selective reporting when including non-randomized studies in systematic reviews on the effects of healthcare interventions. Res Synth Methods. 2013 Mar;4(1):36-47.

61. von Elm E, Altman D, Egger M, Pocock S, Gotzsche P, Vandenbroucke J, et al. The Strengthening the Reporting of Observational Studies in Epidemiology (STROBE) statement: guidelines for reporting observational studies. Journal of Clinical Epidemiology. 2008;61:344-9.

62. Isaia G, Astengo MA, Tibaldi V, Zanocchi M, Bardelli B, Obialero R, et al. Delirium in elderly home-treated patients: a prospective study with 6-month follow-up. Age. 2009 Jun;31(2):109-17.
